# Advances in Contact Tracing: A Bayesian Framework to Improve Network and Transmission Models

**DOI:** 10.1007/s00239-026-10320-9

**Published:** 2026-05-28

**Authors:** Jessica L. Hite

**Affiliations:** Department of Pathobiological Sciences, University of Wisconsin, Madison, 53706 USA

**Keywords:** Network‑informed Bayesian models, Transmission, Contact tracing

## Abstract

Non‑pharmaceutical interventions such as contact tracing, quarantine, and targeted restrictions remain central to outbreak control. Yet, their success depends on understanding how pathogens spread through heterogeneous populations. Here, I highlight an innovative network‑informed Bayesian framework that integrates genomic and contact data across time to reconstruct transmission pathways more accurately (Xu et al. 2026). By modeling network structure as a prior, the approach captures individual‑level heterogeneity and resolves ambiguities that arise when genetic data are sparse . Notably, this framework captures genomic variation using mutation loci rather than complete genomes preserves evolutionary signal while greatly reducing computational demands, and also enables more efficient integration of key metadata. Together, these developments provide a critical step toward designing interventions that more precisely disrupt transmission while minimizing social and economic costs.

The SARS-CoV2 pandemic prompted unprecedented global attention on pandemic preparedness, accompanied by renewed calls to invest in programs to reduce the risk of future outbreaks. Much of this effort has focused on medical countermeasures, vaccines, therapeutics, and diagnostics, designed to limit transmission once a pathogen is already circulating in animal or human populations (Marion et al. 2022; Plowright et al. 2024). These tools are indispensable. Yet, the SARS-CoV2 pandemic also reminded us that long‑standing public health interventions such as contact tracing, quarantine, and restrictions on social interaction remain central to outbreak control, even in an era of rapid vaccine development (Zhang et al. 2021).

These more social interventions carry consequences that extend far beyond epidemiology. Decisions about closing schools, restricting businesses, or limiting social gatherings shape educational trajectories , economic stability, and mental health. , However, years after the acute phase of the pandemic, parents, teachers, business owners, and essential workers continue to seek answers to difficult questions (Celbis et al. 2025). When are closures of businesses and schools warranted? Which contacts matter most? How do we best design more targeted interventions, achieving public health goals while minimizing social and economic costs? Amid an ongoing epidemic of loneliness and social isolation, many also ask whether the sacrifices demanded by distancing policies could be made more effective, or less costly, in the future.

Despite the urgency of these questions, comparatively little attention in global conversations, policy, or funding investments has been devoted to understanding how non‑pharmaceutical interventions — such as contact tracing, quarantine, and targeted restrictions — can be designed to disrupt transmission more efficiently and effectively while also minimizing social and economic costs (Guenduez and Walker 2025; Moran et al. 2025). In the face of the substantial time delays in outbreak detection and response as well as persistent inequities in access to health care, investment in this area is essential to developing efficient, equitable, and cost-effective disease management strategies (Fauci and Folkers 2023). Improving future responses therefore requires a deeper understanding of how pathogens spread through heterogeneous populations, and how interventions can be strategically targeted to the contacts and contexts that matter most (Marion et al. 2022; Swallow et al. (Marion et al. 2022) ).

Recent work by Jianning Xu and co-authors helps advance this endeavor by improving transmission models (Xu et al. 2026). The authors introduce a network‑informed Bayesian framework that integrates genomic and contact network data across time to reconstruct who-infected-whom models. Their approach helps improve both computational efficiency and interpretability. Unlike many standard epidemiological models that assume individuals are, on average, equally susceptible and equally infectious, this framework naturally accommodates individual‑level heterogeneity in transmission by explicitly modeling who can infect whom and under what circumstances. Rather than relying on standard approaches based on genetic similarities in time series data, the model constrains transmission pathways using observed social or spatial networks, allowing infection probabilities to decay with network distance and embedding inference within realistic patterns of human interaction.

This modeling framework also helps address a persistent limitation of transmission models. For many pathogens, particularly those with low mutation rates (many bacteria) or long latency periods (many viruses), genetic and temporal data alone are often insufficient to distinguish among multiple transmission pathways, especially when these pathways operate on similar timescales (Stadler 2009; Volz and Frost 2014; Dawson et al. 2021). These biological factors can obscure the contacts and contexts that drive transmission. In such cases, transmission networks inferred without contact information can remain ambiguous or biologically implausible. By incorporating network structure as a prior, the model allows transmission probabilities to vary based on social and spatial connectivity. More specifically, the model favors transmission pathways that align with observed contact patterns rather than relying solely on genetic distance thresholds. Thus, when genetic and temporal data alone support multiple competing transmission hypotheses, the network prior favors pathways consistent with plausible contact structures, reducing over‑reliance on genetic similarity arising through, for example, purely stochastic processes.

In this way, network‑informed inference makes transmission heterogeneity observable and actionable, helping to provide the resolution needed to translate modeling insights into targeted public health strategies. Mechanistically, incorporating network information does more than improve statistical fit; it changes how uncertainty is accounted for and propagates through the analysis. Additionally, the framework presented here helps improve computational efficiency, enabling efficient information extraction from genomic data. By recording only mutation loci instead of complete genomes, this method captures genomic variation while significantly reducing storage demands.

Together, these innovations provide a three-fold advantage over existing methods. First, they achieve maximal statistical efficiency, i.e., maximum information capture from data. Second, because they allow models to be tailored to specific real or hypothetical transmission networks, they let data speak directly to causal questions of interest. This represents a significant advantage because using empirical data to estimate model parameters in mechanistic models can help reveal cause-effect relationships, rather than mere correlations. Third, they allow integration of genomic data with other data types and sources. In the present context, this means that these methods can be used to assimilate the rich metadata on management practices and other selection pressures.

The broader significance of this framework becomes especially clear when viewed in the context of work on heterogeneous transmission and superspreading. Decades of research have demonstrated that transmission is often highly uneven, with a small fraction of individuals or events accounting for a disproportionate share of onward infections. By reconstructing transmission at the level of individual hosts and their realized contacts, the framework reviewed here provides a concrete way to identify this heterogeneity, rather than assuming equal susceptibility or transmissibility across individuals. In such systems, targeted interventions that focus more on reducing transmission from high‑risk individuals, contacts, or settings can dramatically outperform uniform, blanket measures (Lloyd-Smith et al. 2005; Fauci and Folkers 2023). To realize such strategies, however, those critical pathways must be reliably identified.

Extensive epidemiological evidence indicates that transmission of directly transmitted pathogens is inherently structured by social and spatial networks, even when those networks are imperfectly observed. The framework presented here helps clarify when network‑informed inference works best, and when it does not. The updated model framework outperforms non-network informed approaches, especially when genetic resolution is limited. However, when networks are noisy, incomplete, or only weakly related to actual transmission pathways, incorporating network information can attenuate performance. The current work, therefore, underscores the importance of careful network construction in applied settings: the right type of data with sufficient genetic resolution, key metadata, and sensitivity analyses.

Additionally, using this model to analyze 93 tuberculosis cases revealed that transmission events were more likely to occur through *weak social ties*, casual or infrequent contacts that bridge otherwise disconnected groups, rather than within densely connected clusters. This pattern aligns with Granovetter’s sociological theory, which emphasizes the role of weak‑ties in facilitating the spread of information (Granovetter 1973). In the context of infectious disease, such weak ties may facilitate transmission beyond households or close contact groups, allowing infection to move between distant social circles and geographic areas (Galiwango et al. 2025). These interactions remain constrained by social and spatial proximity, but they occur at the boundaries of tightly connected groups rather than within them, highlighting how transmission can be driven more by weak ties than previously appreciated.

This result has direct implications for public health practice. Interventions focused primarily on strong ties, such as household‑based contact tracing or social distancing practices may systematically miss a substantial fraction of transmission events occurring through casual or transient interactions. Network‑informed models that capture both direct and indirect connections offer a way to identify these critical transmission pathways, helping to explain why some interventions succeed while others fall short.

Taken together, this work also underscores a more fundamental limitation: models are only as powerful as the data that accompany them. In the United States, contact tracing data have historically been collected in fragmented and inconsistent ways, often with substantial gaps that limit rapid integration and inference during outbreaks (Moran et al. 2025). By contrast, several countries have invested more heavily in systematic contact tracing infrastructures. Nations such as South Korea, Taiwan, and Singapore entered the COVID‑19 pandemic with established legal, technical, and public health frameworks for contact tracing, enabling faster identification of transmission chains and more targeted interventions (Kim 2021). The United Kingdom’s experience further illustrates both the potential and the challenges of centralized contact tracing, with periods of improved integration alongside well‑documented limitations (Davis et al. 2021).

Beyond how data are collected, this study highlights the need to rethink *what* data are collected. We now have the technological capacity to quantify pathogen *load* and resolve fine‑scale genetic variation that drives epidemiological and evolutionary processes. The sequencing platforms, computational tools, and trained workforce are increasingly available. Yet, widespread adoption remains constrained by cost and the lack of sustained investments that would reduce these cost barriers. As a result, transmission inference often relies on coarse proxies, such as binary PCR results, rather than the much needed quantitative or genomic data (Moran et al. 2025).

Without sustained investment to close this gap, even the most sophisticated models will remain underutilized. The situation recalls a scene from *Star Trek IV: The Voyage Home*, in which Dr. Leonard “Bones” McCoy, a physician from the "future", encounters twentieth‑century hospital medicine and is appalled by the outdated tools and invasive practices still in use despite the possibility of something far better. Without committing to investing in the data frames and computational tools needed to advance global conversations (Moran et al. 2025; Fauci and Folkers 2023; Alakija 2023), we risk a similar fate, possessing the knowledge and tools to do better, while continuing to practice epidemiology with the equivalent of blunt antediluvian instruments. The work presented here provides an important step forward.

## Data Availability

No datasets were generated or analysed during the current study.
